# Silicone Breast Implant Rupture Triggered by Infection Leading to Skin Ulceration: A Case Report

**DOI:** 10.7759/cureus.75556

**Published:** 2024-12-11

**Authors:** Yoshika Nagata, Shinichi Sezaki, Izumi Kinoshita, Toshihiro Saeki, Takahisa Fujikawa

**Affiliations:** 1 Surgery, Kokura Memorial Hospital, Kitakyushu, JPN; 2 Plastic Surgery, Kokura Memorial Hospital, Kitakyushu, JPN; 3 Pathology, Kokura Memorial Hospital, Kitakyushu, JPN

**Keywords:** breast, implant rupture, silicone breast implant, skin ulceration, surgery

## Abstract

Silicone breast implants (SBIs) are commonly used for breast reconstruction and cosmetic surgery. However, long-term complications associated with SBI, such as rupture and infection, require careful monitoring. Here, we report a case in which coronavirus disease 2019 infection led to immunosuppression and secondary bacterial infection, resulting in skin ulceration and eventual removal of the SBI. This case highlights the potential complications of long-term use of SBI and the need for careful postoperative monitoring.

## Introduction

Silicone breast implants (SBIs) are widely used in breast reconstruction following mastectomy for breast cancer or for cosmetic purposes [1,2]. Long-term complications such as implant rupture, capsular contracture, and foreign body granuloma are of great concern and may be exacerbated by systemic conditions such as infection or rupture [3,4]. Recently, new complications have been reported. These include breast implant-associated anaplastic large cell lymphoma (BIA-ALCL) [5]. Evaluation with appropriate imaging is important for the early detection of these complications.

The novel coronavirus disease 2019 (COVID-19), identified in 2019, has evolved into a global pandemic, with the initial case confirmed in Japan in January 2020 [6,7]. Reports indicate elevated severity and mortality rates, along with various sequelae that endure postinfection. An international registry study indicates that the severity of the disease may affect the range of skin manifestations associated with COVID-19, including ulcers, purpura, necrosis, nonspecific erythema, morbilliform eruption, pernio-like lesions, and vesicles [8,9]. The effects of COVID-19 infection and COVID-19 mRNA vaccination on SBI have been reported, but a direct association has not been confirmed [10]. We herein report a case of SBI rupture, which started as inflammation of the breast skin during a COVID-19 infection, gradually progressed to ulceration, and ultimately required surgical intervention due to the SBI infection.

## Case presentation

**Figure 1:**
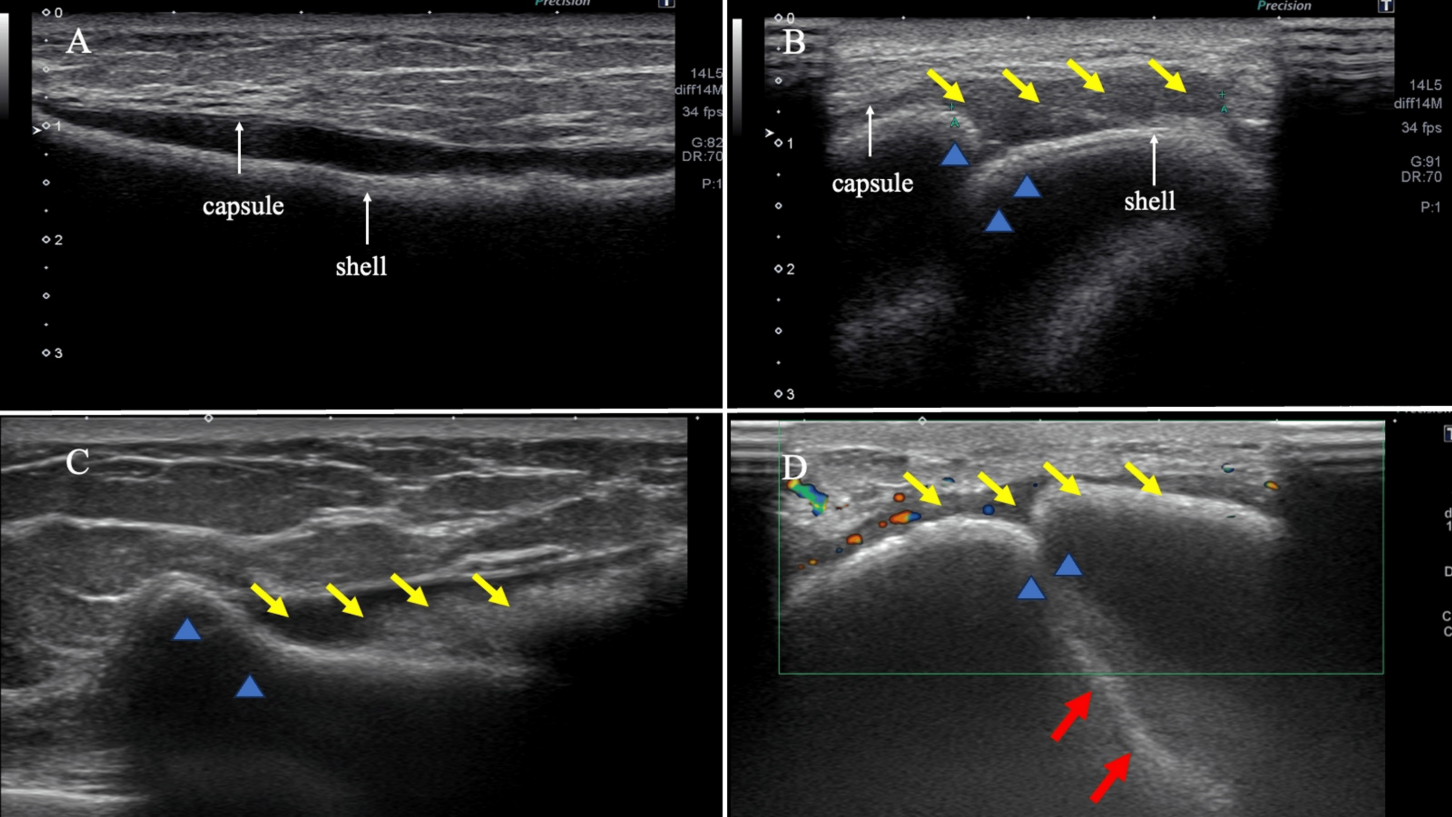
US findings three years ago (A,B) and at presentation (C,D) (A) US examination of the right breast showed separation of the outer shell and the capsule (white arrow). There was no increase in the internal echo level. (B) The left breast exhibited shell deformation (blue arrowhead) of the SBI, capsule separation from the shell, and a minor increase in subcapsular density (yellow arrow). (C) The US of the right breast at presentation showed shell deformation (blue arrow), capsule-shell separation, and a slight increase in subcapsular density (yellow arrow). (D) SBI showed hyperechoic images (red arrow) in the silicone, suggesting an extracapsular fracture in the left breast US: ultrasonography; SBI: silicone breast implant

A 43-year-old Japanese woman noticed a serous nipple discharge from her right breast and visited our hospital three years ago. She was taking oral medication for a psychiatric disorder, but this was well-controlled, and she had no significant family history. Breast cancer screening revealed no malignant lesions in her breast. Although she had no history of breast trauma, she had undergone bilateral mammoplasty with SBIs for cosmetic reasons 20 years ago. The ultrasonography (US) of the right breast revealed a separation between the capsule and the shell, with mild waviness, but no increase in echo density within the capsule, leading to the diagnosis of mild SBI deformation. The left breast showed shell deformation of the SBI, separation of the capsule from the shell, and a slight increase in subcapsular density. The findings were suggestive of minor damage; however, it was concluded that the damage was limited to the capsule (Figures 1A, 1B). She, therefore, wished to have them removed at her own expense and had written a request after the SBI assessment, but she had not seen a plastic surgeon for personal reasons.

During the COVID-19 pandemic, the patient was diagnosed with COVID-19 virus infection based on a positive COVID-19 antigen test. Although she had not received the COVID-19 vaccine, her infection was mild. She experienced upper respiratory symptoms, a high fever, and noticed redness under her left breast. The red area grew larger and became more painful; the skin began to break down, and the bleeding and discharge persisted. Three months after her COVID-19 infection, the patient returned to our hospital. We observed the resolution of upper respiratory symptoms, and no other clinical signs were suggestive of immune abnormalities. During the physical examination, we noted a skin ulcer on the inferomedial side of the left breast, which exposed the SBI. Bacterial cultures of the exudate showed *Staphylococcus aureus*. Laboratory analysis showed a slightly elevated white blood cell count of 9.6 (reference value: 3.0-8.9) × 10^3^/μL, neutrophils of 71.8% (44%-73%), platelet of 39.5 (12.0-39.0) × 10^3^/μL, and C-reactive protein of 2.3 (0.0-0.5) mg/L. The blood glucose level was 100 mg/dL, and the glycosylated hemoglobin (National Glycohemoglobin Standardization Program) was 5.6%. The antinuclear antibody was negative, within the normal range (≤1:40). The level of soluble interleukin-2 receptor was 234 (121-613) U/mL. These data are shown in Table 1.

**Table 1:** Laboratory findings WBC: white blood cell; CRP: C-reactive protein; HbA1c: glycosylated hemoglobin; NGSP: National Glycohemoglobin Standardization Program; ANA: antinuclear antibody; sIL-2R: soluble interleukin-2 receptor

Parameter	Result	Reference range
WBC	9.6 × 10^3^/uL	3.0-8.9 × 10^3^/uL
Hemoglobin	11.3 g/dL	11.57-15.9 g/dL
Platelet	39.5 × 10^3^/μL	12.0-39.0 × 10^3^/μL
Neutrophils	71.8%	44%-73%
Lymphocytes	21.4%	19%-47%
Monocytes	4.5%	2.0%-8.0%
Eosinophils	1.9%	1.0%-9.0%
Basophils	0.4%	0.0%-2.0%
CRP	2.3 mg/L	0.0-0.5 mg/L
Glucose	100 mg/dL	70-109 mg/dL
HbA1c (NGSP)	5.6%	4.6%-6.2%
ANA	Negative	≤1:40
sIL-2R	234 U/mL	121-613 U/mL

The US of the right breast showed shell deformation of the SBI, separation of the capsule and shell, and a slight increase in subcapsular density. The left breast was suspected to be fractured outside the capsule as the SBI showed severe deformation and hyperechoic images within the silicone (Figures 1C, 1D). Cortical thickening was present in the left axillary lymph node, and fine-needle aspiration cytology revealed a reactive lymphadenopathy.

**Figure 2:**
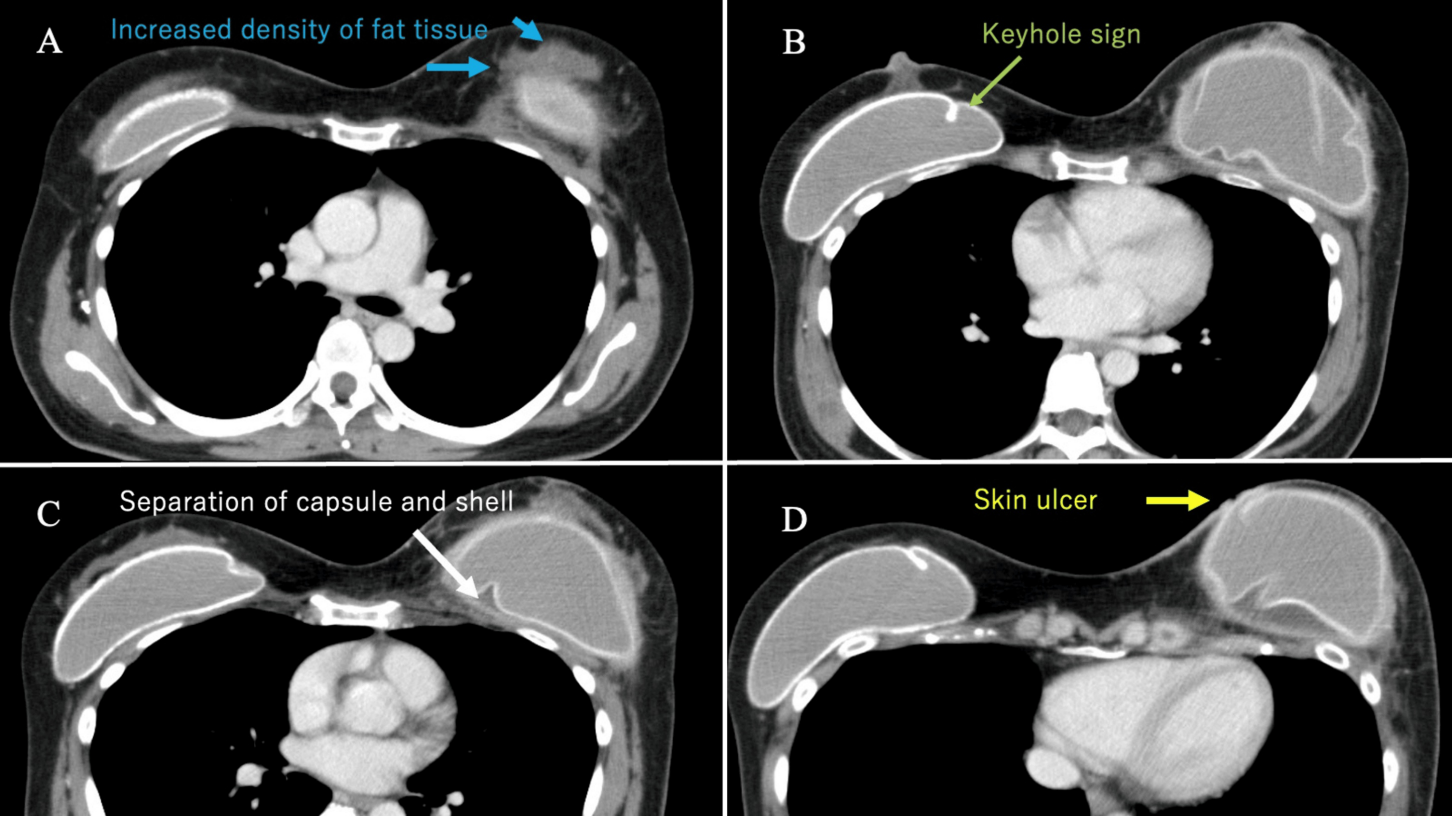
Contrast-enhanced CT findings, showing (A) increased density of adipose tissue around the SBI (blue arrows), (B) keyhole sign (green arrows) within both SBIs, (C) separation of capsule and shell (white arrows), and (D) skin ulcers (yellow arrows) CT: computed tomography; SBI: silicone breast implant

Computed tomography showed keyhole signs within both SBIs. The skin of the left breast had collapsed, and the density of the fat around the SBI had increased (Figure 2).

**Figure 3:**
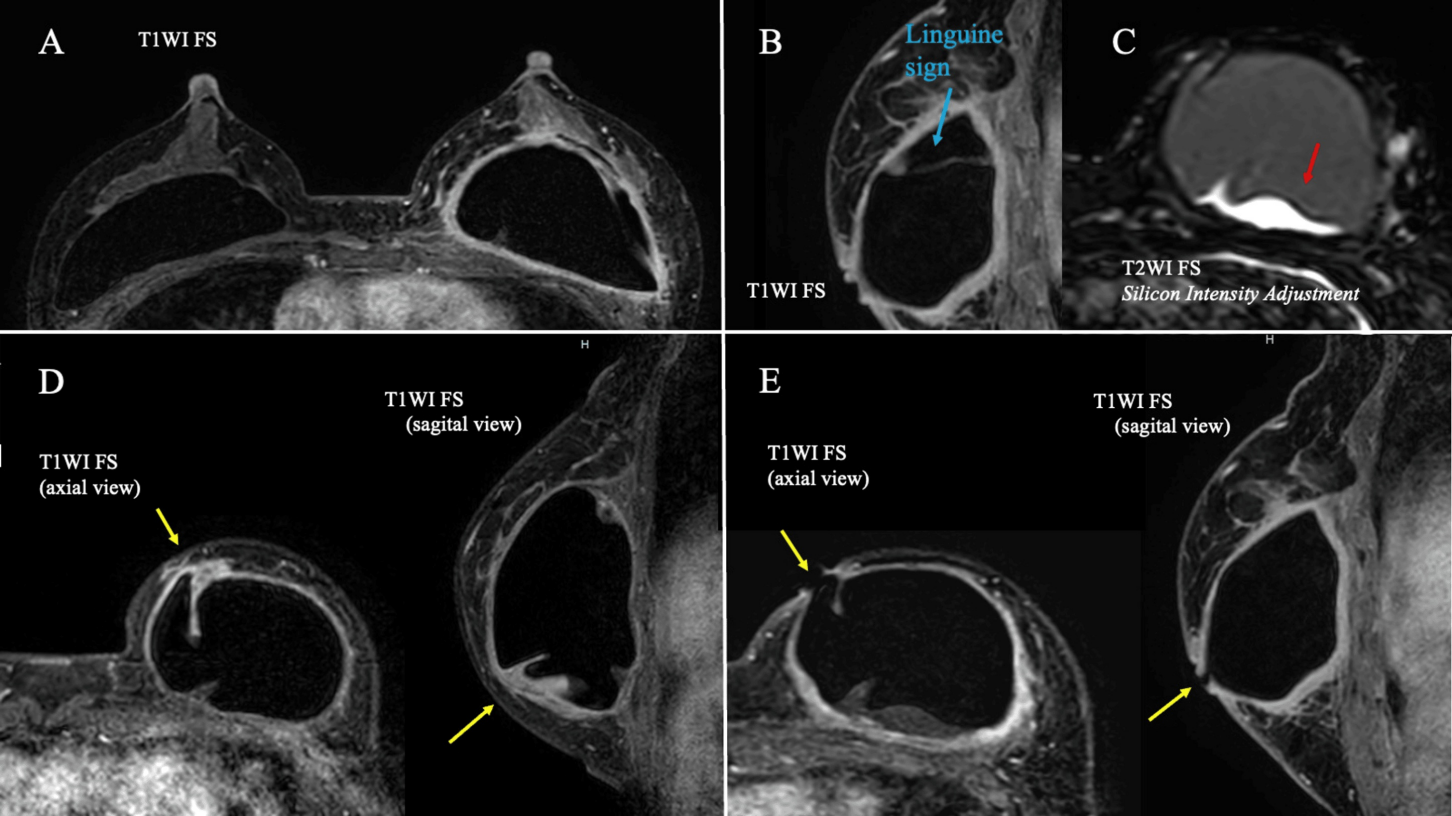
MRI findings. (A) A contrast-enhanced MRI showed enhancement around the SBI. (B) The sagittal view using T1WI with FS showed the linguine sign (blue arrow). (C) The silicone-enhanced T2WI image showed an area of high signal within the SBI (red arrow). (D) The site of the skin ulcer was observed three years ago, showing deformation of the SBI (yellow arrow). (E) Silicone was exposed at the site of the skin ulcer (yellow arrow) FS: fat suppression; MRI: magnetic resonance imaging; SBI: silicone breast implant; WI: weighted image

Contrast-enhanced magnetic resonance imaging (MRI) showed deformation of the SBI in the left breast and severe enhancement of the capsule on T1-weighted imaging with fat suppression. The signal within the SBI was also heterogeneous, suggesting extracapsular damage. Upon comparing the location of the skin ulcer in the left breast with the MRI image from three years ago, the sagittal and axial views confirmed the rippling of the SBI (Figure 3).

**Figure 4:**
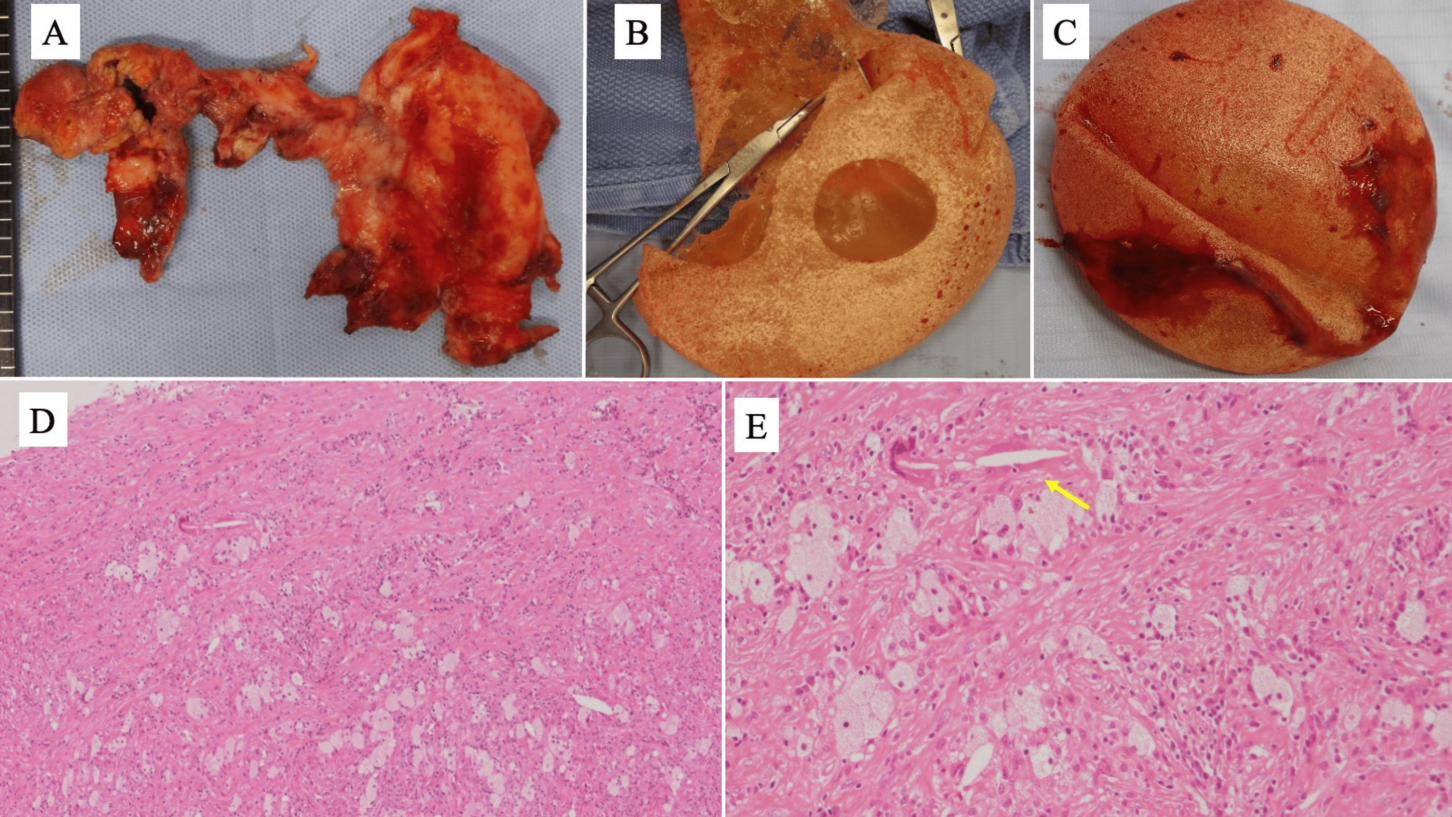
Macroscopic findings of surgical specimens, excised SBI, and pathologic findings of the capsule. (A) Macroscopic findings of thickened capsule. (B) The excised SBI of the left breast became friable over time and progressed to extracapsular damage. (C) The excised SBI of the right breast revealed findings that were consistent with superficial silicone gel leakage. (D) HE staining of the excised capsule revealed a composition of a hyalinized fibrous stroma with a strong inflammatory response (40×). (E) Inflammatory findings such as granulation tissue and cholesterol clefts (yellow arrow) were observed (100×) SBI: silicone breast implant; HE: hematoxylin and eosin

The above findings led to the diagnosis of a mild injury in the right SBI, an extracapsular injury in the left SBI, and the planning of surgical treatment. Surgery revealed a mild injury to the right SBI with silicone gel leaking from the surface. The left side had an extracapsular injury and severe capsular contracture, and then the capsule was resected (Figures 4A-4C). The resected capsule was composed of hyalinized fibrous stroma, and inflammatory reactions, hemorrhage, granulation tissue, and cholesterol clefts were observed. Inflammatory cell infiltration was also observed in the surrounding adipose tissue (Figures 4D, 4E). There were no obvious malignant findings in the capsule. Furthermore, the cytology of the exudate did not reveal any malignant cells, thus excluding the possibility of BIA-ALCL. We resected the SBI without reconstruction to control infection, but the wound was good, and no complications, such as silicone granuloma, occurred.

## Discussion

SBIs are frequently used for cosmetic or breast reconstruction after mastectomy due to breast cancer [1,2]. For cosmetic purposes, an SBI is placed under the mammary gland and above the pectoralis major muscle. In contrast, breast reconstruction for breast cancer often involves placing an SBI below the pectoralis major muscle to prevent damage. Long-term complications such as implant rupture, capsular contracture, and foreign body granuloma are of great concern and may be exacerbated by systemic conditions such as infection and rupture [3,4]. The estimated incidence of SBI rupture ranges from 8.7% to 24.2% over 10 years. SBI rupture is also classified according to the degree of rupture: shell rupture, intracapsular rupture, and extracapsular rupture. Many patients remain asymptomatic after SBI rupture, so imaging screening for asymptomatic implant rupture is necessary. Characteristic imaging findings of rupture include the keyhole sign, linguine sign, and subscapular line sign, and MRI has been devised to adjust the intensity of silicone depending on the case. MRI has been reported to be effective in detecting SBI rupture as it has a higher sensitivity for rupture compared to the US [4]. Furthermore, new complications such as BIA-ALCL have been reported in recent years [5]. BIA-ALCL was classified as a rare malignancy by the World Health Organization in 2016. The majority of cases present with a delayed effusion or mass. Appropriate imaging is important for early detection of these SBI-related complications. Screening for recurrence and metastasis is recommended for breast cancer patients for 10 years after surgery in Japan, but there are no guidelines for regular screening of cosmetic breast reconstructions or long-term follow-up cases.

The novel COVID-19 has become a global epidemic known as a pandemic in just a few months since the first case was reported in early December 2019 [6]. In Japan, the first case was confirmed in January 2020. The disease has a high rate of severe illness and mortality, and after-effects have been reported even after infection [7]. It has been reported that there are five main types of skin disorders that occur as a result of the novel coronavirus: urticaria, erythema-papular rash, vesicles, livedo reticularis, and COVID toe. A study from an international registry reveals a wide range of cutaneous manifestations associated with COVID-19, including morbilliform, pernio-like, urticarial, macular erythema, vesicular, papulosquamous, and retiform purpura [8]. An analysis of patterns of mucocutaneous disease in 296 adult COVID-19 patients suggested that severity and rash symptoms may be related [9].

The effects of COVID-19 infection and COVID-19 messenger RNA vaccination on SBI were investigated. Cases have been reported in which capsular contracture and delayed seroma occurred after vaccination, requiring surgical intervention such as capsulectomy and SBI removal [10]. These vaccine side effects are thought to be due to a proinflammatory, profibrotic dose-response response and activation of angiotensin-converting enzyme 2, but the association between SBI complications and the vaccine has not been demonstrated [11]. COVID-19 infection causes a variety of systemic symptoms, including vasculitis, vasculitis-like processes, and coagulopathy. There was a case report of a patient in the severe category of COVID-19 who developed a sterile abscess in the breast due to vasculitis seven months after infection and recovered with abscess drainage alone without the need for SBI removal [12]. Another case report describes a patient with mild COVID-19 who underwent surgery to excise a seroma that developed late during the infection [13].

This case shows that systemic infections such as COVID-19 can exacerbate long-term complications associated with SBI. It is highly likely that the SBI deformation and mild inflammation present three years ago contributed to the development of skin ulceration due to COVID-19 infection, which triggered the detection of implant rupture. Cortical thickening of the axillary lymph nodes post-COVID-19 vaccination is now acknowledged as a prevalent adverse effect [14]. In this case, a fine needle aspiration biopsy was performed on the enlarged axillary lymph node and was found to be reactive. Another well-known cause of axillary lymphadenopathy in SBI is silicone lymphadenopathy [15]. In addition, cytology and capsule histology ruled out the possibility of BIA-ALCL. SBI rupture can cause various complications such as deformation, pain, redness, and foreign body granuloma formation, but fortunately, there was no evidence of foreign body granuloma outside the breast in the current case.

## Conclusions

We present a case of SBI rupture resulting from breast skin inflammation that ulcerated and caused SBI infection, requiring surgical treatment. This case highlights the need for careful long-term follow-up for patients with SBI. As systemic infections such as COVID-19 weaken the immune system, attention should be paid to infections of artificial materials, including implants. The definitive treatment for ruptured silicone implants remains surgical treatment, and in this case the patient progressed well after removal.
